# Increased Expression of DNA2 Was Linked to Poor Prognosis in Breast Cancer

**DOI:** 10.1155/2021/8860728

**Published:** 2021-01-26

**Authors:** Yingyan Han, Zeyu Zhang, Zhi Wang, Shujuan Sun

**Affiliations:** Cancer Biology Research Center (Key Laboratory of the Ministry of Education), Tongji Hospital, Tongji Medical College, Huazhong University of Science and Technology, Wuhan, China

## Abstract

DNA double-strand break (DSB) repaired by homologous recombination (HR) is an essential process for breast cancer cells to survive. DNA2 nuclease acts parallel to homologous recombination (HR). Here, we investigated the detailed clinical attribute of DNA2 in breast cancer and the role of DNA2 in breast cancer cells' growth. We found that elevated expression of DNA2 was obviously linked to poor prognosis in breast cancer. Further, DNA2 expression was increased in the ER-negative group, PR-negative group, HER2-positive group, and high-grade group via analyzing 2,509 breast cancers in “cBioportal” and 3,063 breast cancer data in “bc-GenExMiner.” Besides, the immunohistochemical staining in 26 breast cancer tissues also showed that elevated expression of DNA2 was correlated with ER-/PR-/HER+. To further detect the role of DNA2 in breast cancer cells, we took GESA, GO, and KEGG analyses and found that DNA2 was enriched in cell cycle and DNA replication pathways. Furthermore, silencing of DNA2 inhibited cell growth in T47D and MD-MB-231 breast cancer cells and suppressed tumor growth *in vivo*, indicating DNA2 functioned importantly in breast cancer progression and maybe a potential prognostic marker in breast cancer. Our research reveals that DNA2 is a biomarker for diagnosis and prognosis in breast cancer from multiple perspectives and gives a new clue for further preclinical and clinical investigation.

## 1. Introduction

Despite significant advances in our understanding and management of breast cancer over the past 50 years, the disease remains a serious health problem and a major challenge on a global scale [1, 2]. According to the expression status of three receptors: the estrogen receptor (ER), progesterone receptor (PR), and human epidermal growth factor receptor (HER2), breast cancer has been classified into Luminal A (LumA), Luminal B (LumB), epidermal growth factor receptor ERBB2/HER2-overexpressing (HER2+), and basal epithelial-like (BL). This can be helpful to predict patient outcomes and provide new choices for treatment [3–5]. In current practice, treatment options for breast cancer patients consist of surgery, chemotherapy, radiotherapy or targeting of classical markers of breast cancer subtype, and emerging new therapy [5]. However, these treatments have some limitations, and new studies are needed [3, 6, 7]. So uncovering and improved understanding of novel biomarkers could effectively contribute to improving patient stratification and treatment.

Analyses of human tumors have demonstrated that the presence of DNA damage, particularly DNA double-strand breaks (DSB), distinguishes precancerous lesions and cancer from normal tissues [8]. DSBs are the most dangerous type of DNA damage, as a single unrepaired DSB can trigger apoptosis. DNA replication helicase/nuclease 2 (DNA2) is a mediator of genome stability that is required for proper Okazaki fragment maturation, for DSB repair, and for protecting stalled replication forks [9, 10]. Also, DNA2 provides cells with survival advantages under conditions of replication stress via facilitating homologous recombination to repair replication-associated DNA DSBs [11]. Thus, DNA2 high expression may promote the proliferation of cancer cells, and targeting DNA2 may be a new cancer therapy [12–14]. It has recently been found that DNA2 is highly expressed in breast cancer tissues [11, 15]. However, we need much more details of DNA2, such an important gene, in clinical relevance and application value with breast cancer.

Here, we found elevated DNA2 expression was closely associated with poor prognosis in breast cancer. Our study indicated DNA2 expression was increased in the ER-negative group, PR-negative group, HER-positive group, and the higher pathological grade group. Results showed that inhibition of DNA2 suppressed tumor growth. These findings improved the understanding of DNA2, providing evidence that DNA2 was a potential biomarker of diagnosis and prognosis in breast cancer.

## 2. Materials and Methods

### 2.1. Cell Culture and Transfection

Breast cancer cell lines MDA-MB-231 and T47D were got from the American Type Culture Collection (ATCC). T47D were grown in RPMI-1640 basal medium containing 10% FBS at 37°C with 5% CO_2_. MDA-MB-231 cells were grown in L-15 medium containing 10% FBS in free gas exchange with atmospheric air.

### 2.2. The Clinical Attribute Analysis

We used data contained at cBioportal (http://www.cbioportal.org) [16] and “breast cancer Gene-Expression Miner” (bc-GenExMiner, http://bcgenex.centregauducheau.fr/) [17].

### 2.3. Outcome Analysis

Both the KM Plotter Online Tool (http://www.kmplot.com) and bc-GenExMiner (http://bcgenex.centregauducheau.fr/) were used to analyze the relationship between the DNA2 expression and patient clinical outcome in breast cancer [18].

### 2.4. DNA2 Immunohistochemistry

Manual immunohistochemical staining was performed in order to determine DNA2 expression, using an anti-DNA2 antibody (1 : 150 dilution, Proteintech, China). A thoracic pathologist scored DNA2 staining by multiplying the intensity (0–3+) and extent (0–100%) of staining via light microscopy (range 0–12).

### 2.5. Cell Counting Kit-8 (CCK8) Assay

Cells were seeded at 5,000 cells per well in 96-well plates. Cells were allowed to adhere for 12 h, 24 h, 36 h, and 48 h. Then, each well was incubated with 10 *μ*l CCK8 reagents for 2 h in the incubator. The OD value of each well was measured at 450 nm using a microplate reader.

### 2.6. Function Analysis

Gene expression profiles of breast cancer patients were divided into high and low expression groups according to the median value of expression of DNA2. GSEA was used to detect the potential mechanism of DNA2 expression on breast prognosis. Gene set permutations were performed 1,000 times for each analysis. Gene sets with a *p* value <0.05 and false discovery rate (FDR) <0.05 were regarded as significantly enriched. GO analysis was performed using Enrich GO function in cluster Profiler R package 6, with the following parameters: ont = ^“^all,^”^*p* value cutoff =0.05, and *q* value cutoff =0.05. KEGG analysis was performed by Enrich KEGG function of R package “cluster Profiler,” with the following parameters: key Type = ^“^kegg,^”^*p* value cutoff =0.05, and *q* value cutoff =0.05.

### 2.7. Western Blot Analysis

Western blot analysis was performed according to the manufacturer's instructions (http://www.ptgcn.com/media/1474/wb-collection_for-web.pdf).

### 2.8. Antibodies

DNA2 antibody (catalog number: 18727) and SDHA antibody (catalog number: 14865) were purchased from Proteintech. *γ*H2A was purchased from Abcam (catalog number: ab2893).

### 2.9. Transfection

The siRNAs were transfected into tumor cells using Lipofectamine 3000 according to standard protocols. The siRNA sequences were as follows: siDNA2-1 TGGTGAGGATTGGTTTCAT, siDNA2-2 CACTAGAACACTGGCATTG, siDNA2-3 UACCGCUUAAAUCUAAGUCAAdTdT; shDNA2#1: CCGGACCTGGTGTTGGCAGTCAATACTCGAGTATTGACTGCCAACACCAGGTTTTTTTG.

### 2.10. Use of Animals

All severe combined immunodeficiency (SCID) mice were got from Beijing HFK Bioscience Co., Ltd. (Beijing, China), and experiments were approved by the Committee on the Ethics of Animal Experiments of Tongji Medical College. The mice were kept in the accredited animal facility of Tongji Medical College.

### 2.11. Statistics

Data is presented as the mean ± s.d. Statistical comparisons between groups were analyzed using Student's *t*-test.

## 3. Result

### 3.1. High mRNA Level of DNA2 Predicts Poor Prognosis

**Figure 1:**
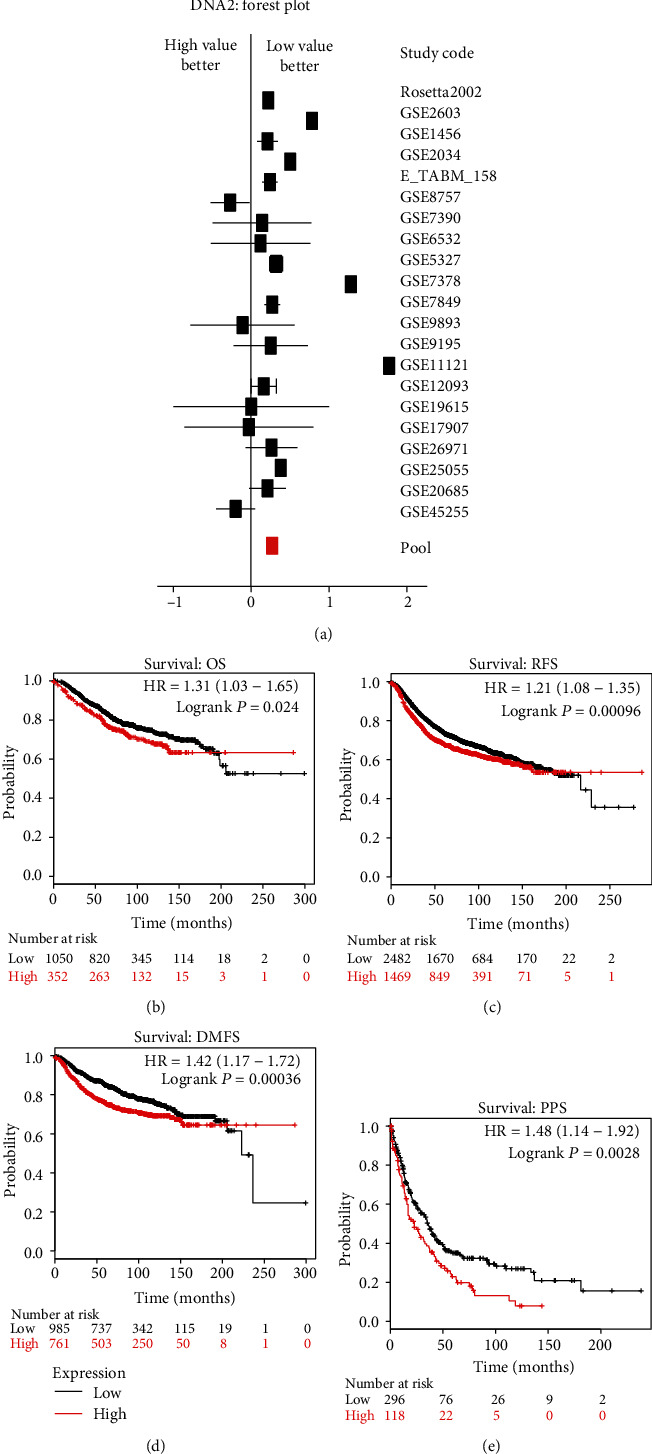
High expression of DNA2 predicted the worse outcome of breast cancer patients. (a) The forest plot indicated DNA2 univariate Cox analysis. The red box represented the hazard ratio (HR, HR = 1.22, *p* < 0.0001) of “Pool” which consists in merging all cohorts pooled together with data from all studies previously converted to a common scale with a suitable normalization. Event status: metastatic relapse (MR). (b–e) Determination of prognostic value of DNA2 mRNA expression in KM plotter database (http://kmplot.com/analysis/index.php?p=service&cancer=breast). The Affymetrix ID is valid: 213647_at (DNA2). Survival curves were plotted for all breast cancer patients with different survival events. (b). Survival: overall survival (OS), *n* = 1,402. (c) Survival: relapse-free survival (RFS), *n* = 3951. (d) Survival: distance metastasis-free survival (DMFS), *n* = 1,746. (e) Survival: postprogression survival (PPS), *n* = 414.

Genetic and molecular studies demonstrate that the highly conserved DNA2 nuclease/helicase plays a crucial role in counteracting replication stresses and was correlated with poor prognosis in estrogen receptor-positive patients [11, 19]. To systematically test the association between DNA2 expression and patient survival in breast cancer, both the bc-GenExMiner and KM plotter were performed. As shown in Figure 1(a), DNA2 univariate Cox analysis with metastatic relapse (*n* = 3,509) from bc-GenExMiner showed that DNA2 was a key gene, and high DNA2 expression was found to indicate poor prognosis in 7 of 21 studies (HR > 1, *p* < 0.1). And merging all studies pooled together with data from all studies previously converted to a common scale with a suitable normalization suggested that high expression of DNA2 indicated a poor prognosis (HR > 1, *p* < 0.0001, Figure 1(a)). Similar results were got from the DNA2 univariate Cox analysis with any event information (metastatic or any relapse, or death) (*n* = 4,706, data not shown).

Further, we used the KM plotter and determined the prognostic value of DNA2. The Affymetrix ID is valid: 213647_at (DNA2). The survival curves obtained from the KM plotter suggested that DNA2 high expression was correlated to worse overall survival (OS) for all breast cancer patients, hazard ratio (HR) = 1.31(1.03 − 1.65), *p* = 0.024 (Figure 1(b)). Also, for all breast cancer patients, high mRNA level of DNA2 was correlated to worse relapse-free survival (RFS) (HR = 1.21, *p* = 0.00096), or worse distance metastasis-free survival (DMFS) (HR = 1.42, *p* = 0.00036), or worse postprogression survival (PPS) (HR = 1.48, *p* = 0.0028) (Figures 1(c)–1(e)).

### 3.2. Association between DNA2 Expression and Clinicopathologic Characteristics in Breast Cancer

**Figure 2:**
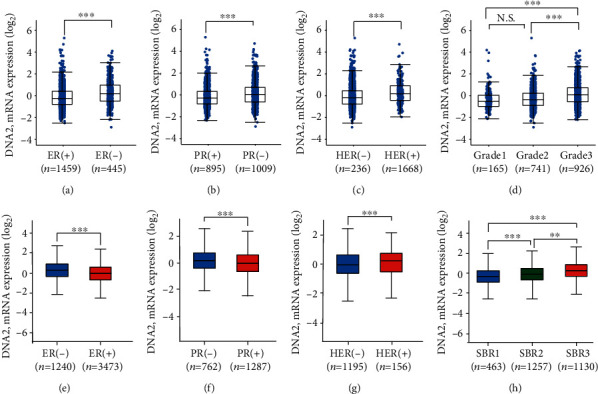
The clinical significance of DNA2 in breast cancer. (a–d) The data of DNA2 was obtained from Breast Cancer (METABRIC, Nature 2012 & Nat Commun 2016) in the cBioPortal for Cancer Genomics (http://www.cbioportal.org). The correlation between the mRNA expression of DNA2 and ER status (a), PR status (b), HER status (c), and neoplasm histologic grade of breast cancer (d). (e–h) The data of DNA2 was obtained from bc-GenExMiner (http://bcgenex.centregauduheau.fr/). The association between DNA2 mRNA level (Log2) and ER Status (e), PR status (f), HER status (g), Scarff Bloom & Richardson grade (SBR, H). ^∗^*p* < 0.05, ^∗∗^*p* < 0.001, ^∗∗∗^*p* < 0.0001.

To obtain a more accurate clinical significance of DNA2 in breast cancer, we analyzed the RNA expression of breast cancer using mRNA expression *z*-score (U133 microarray only) data available from the open-access website (http://www.cbioportal.org/). The genome profiles of 2,509 breast cancers were used to analyze the association of expression of DNA2 in breast cancer with clinical characteristics [20]. The results suggested that the mRNA expression of DNA2 was higher in the ER-negative group (Figure 2(a)), PR-negative group (Figure 2(b)), HER-positive group (Figure 2(c)), and the high-grade pathological group (Figure 2(d)). As bc-GenExMiner (http://bcgenex.centregauducheau.fr/) included molecular subtyping of 3,063 breast cancers, we also used bc-GenExMiner to analyze the correlation between DNA2 expression and receptor pattern. As expected, the analogous result was got from bc-GenExMiner (Figures 2(e)–2(g)). Further, the higher pathological grade also showed higher expression of DNA2 (Figure 2(h)). It was worth mentioning that DNA2 expression in the breast cancer cohort from bc-GenExMiner was obviously lower in Luminal A breast cancer than in the other subtypes (Figure S1A), while DNA2 expression in the breast cancer cohort from cBioportal was obviously lower in ER+/HER2-low proliferation breast cancer than in the other subtypes (Figure S1B).

**Figure 3:**
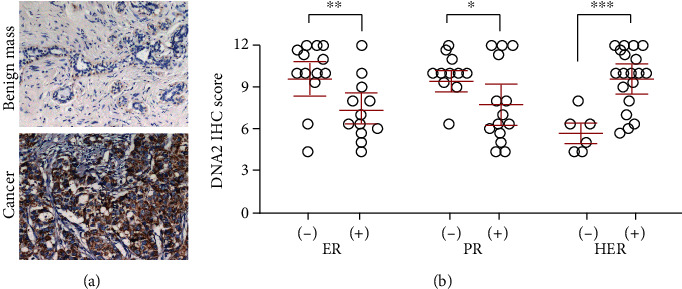
Clinical relevance of DNA2 in breast cancer obtained via IHC. (a) DNA2 protein expression in breast cancer and breast benign tissues. (b) The statistic chart showed the relation between DNA2 protein expression and ER status, PR status, and HER2 status. ^∗^*p* < 0.05, ^∗∗^*p* < 0.001, ^∗∗∗^*p* < 0.0001.

Given the clinical attribute of DNA2 mRNA expression in breast cancer, we sought to assess the relationships between DNA2 protein expression and clinicopathologic indicators via immunohistochemical (IHC) staining in 26 breast cancer tissues and 7 breast benign tissues (the clinical characteristics is shown in Table S1). As shown in Figure 3(a), DNA2 protein-positive staining was located in the cytoplasm and upregulated in breast cancer tissues. Further, the analysis of the relation between DNA2 expression and ER status indicated that DNA2 protein level was higher in the ER-negative group (Figure 3(b)). Analogously, the PR-negative group and the HER-positive group also showed an increased DNA2 protein level (Figure 3(b)).

### 3.3. Knockdown of DNA2 Inhibits Breast Cancer Cells Growth *In Vitro*

**Figure 4:**
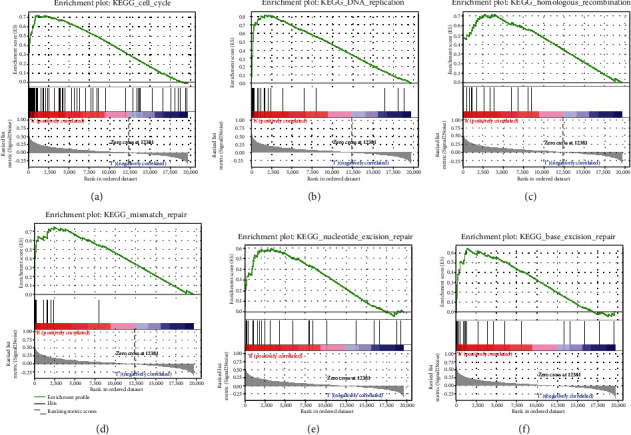
The GSEA analysis of DNA2.

**Figure 5:**
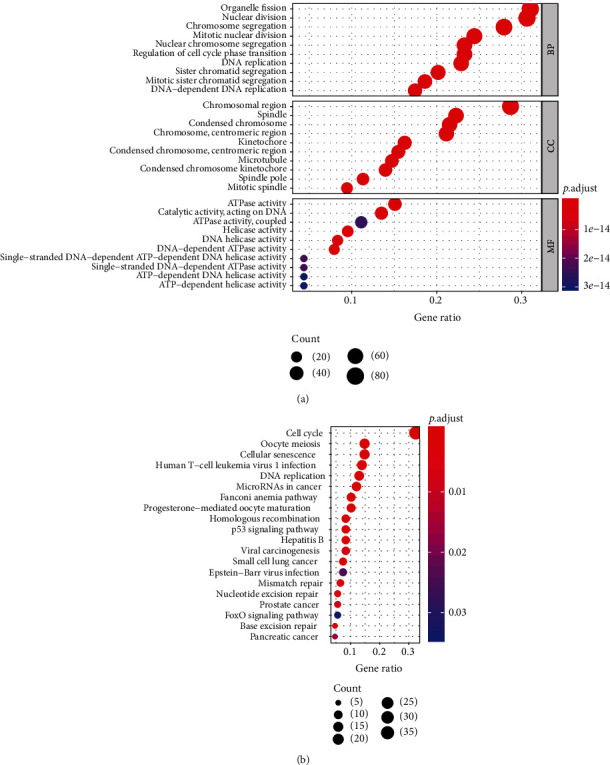
GO and KEGG analysis of DNA2. (a) GO (Gene Ontology) enrichment analysis of differentially expressed DNA2 genes. (b) KEGG enrichment analysis of differentially expressed DNA2 genes.

GSEA was performed to evaluate hallmark effect gene sets and KEGG signaling pathway gene sets, which were associated with upregulated DNA2 in the TCGA breast cancer samples. Nineteen signaling pathways involved in the cell cycle, oocyte meiosis, progesterone-mediated oocyte maturation, basal transcription factors, RNA degradation, spliceosome, DNA replication, ubiquitin-mediated proteolysis, homologous recombination, mismatch repair, pyrimidine metabolism, nucleotide excision repair, base excision repair, p53 signaling pathway, aminoacyl tRNA biosynthesis, purine metabolism, cysteine and methionine metabolism, lysine degradation, and one carbon pool by folate were differentially enriched in the high expression phenotype of DNA2 (Table 1). Six signaling pathways that may be closely connected to the cell growth of breast cancer are shown in Figures 4(a)–4(f). According to cluster profile GO and KEGG analyses, we found that the cell cycle, DNA replication, homologous recombination, mismatch repair, nucleotide excision repair, and base excision repair pathways were significantly enriched in samples with high DNA2 expression (Figures 5(a) and 5(b)).

**Figure 6:**
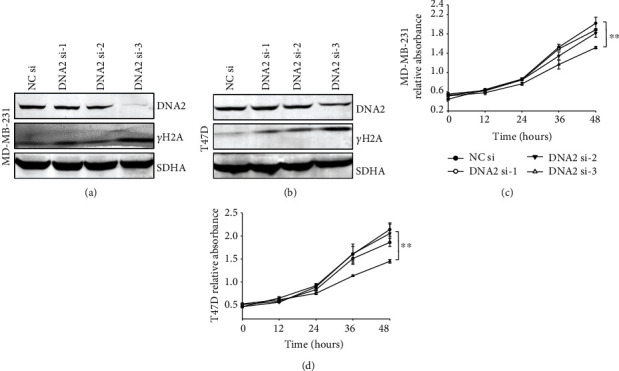
Knockdown of DNA2 inhibits breast cancer cell growth. (a, b) MD-MB-231 cells (a) and T47D cells (b) were transfected with indicated siRNAs, and the protein expression was analyzed with indicated antibodies. (c, d) MD-MB-231 cells (c) and T47D cells (d) were transfected with indicated siRNAs. Equal numbers of cells were plated in 96-well plates in triplicate, and viable cell proliferation was assessed using CCK-8 assays (^∗∗^*p* < 0.01, Student's *t*-test).

To further assess the effect of DNA2 on cell growth of breast cancer, MD-MB-231 cells and T47D cells were transfected with three pairs of siRNA which selectively target DNA2. As shown in Figures 6(a) and 6(b), the third pair siRNA DNA2 si-3 effectively inhibited the expression of DNA2, which resulted in the increase of *γ*H2A, a marker of DNA damage. Further, treatment with DNA2 si-3 inhibited cell growth in MD-MB-231 cells and T47D cells (Figures 6(c) and 6(d)).

### 3.4. Silencing of DNA2 Leads to Inhibition of Tumor Growth *In Vivo*

**Figure 7:**
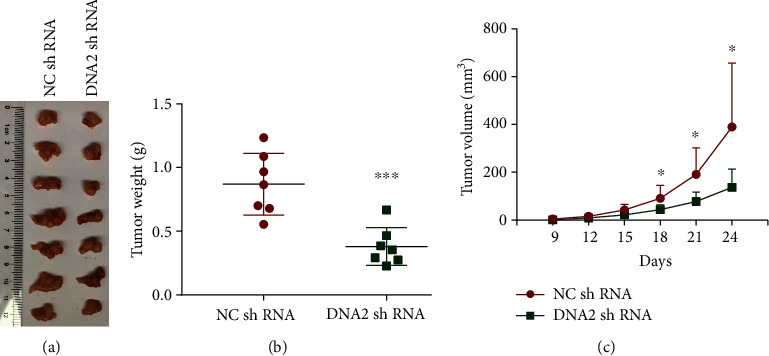
Effects of DNA2 expression on tumor growth *in vivo*. A total of 2 × 10^6^ MD-MB-231 cells expressing DNA2 shRNA or NC shRNA were subcutaneously injected into SCID mice (*n* = 7 per group). (a) Tumors were excised at 28 days after injections and photographed. (b) Tumor weights were measured. Data show the means and were analyzed using Student's *t*-tests. (c) Tumor volumes (mm^3^) were estimated using calipers for 24 days after tumor cells injection. ^∗^*p* < 0.05, ^∗∗∗^*p* < .001, Student's *t*-test, *n* = 7.

To further determine if suppression of DNA2 affects tumor growth *in vivo*, we injected MD-MB-231 cells stably transfected with NC shRNA or DNA2 shRNA into severe combined immunodeficiency (SCID) mice and then monitored tumor growth by measuring subcutaneous tumors periodically. Consistent with previous results, knockdown of DNA2 led to the marked decrease of xenograft tumor growth (Figures 7(a)–7(c)).

## 4. Discussion

DNA2 is reported upregulated in several cancer types and facilitates homologous recombination to repair replication-associated DNA DSBs, thereby providing cancer cells with survival advantages under replication stress [11, 21]. In our study, we found increased expression of DNA2 in breast cancer was closely associated with ER (-), PR (-), HER (+), and predicted poor prognosis. Moreover, we also screened DNA2-related signaling pathways in breast cancer to understand the potential mechanism involved in the regulation of breast cancer development by DNA2.

Breast cancer is considered one of the most dangerous diseases in women's health. There is an article that refers to the high expression of DNA2 in breast cancer predicts worse overall survival based on a study on 295 breast cancer cases. However, the small sample size leads to the sampling error in independent research, which weakens its credibility [11]. In our study, we took a more detailed and much deeper study on DNA2 in breast cancer. DNA2 univariate Cox analysis with metastatic relapse (*n* = 3,509) from bc-GenExMiner showed that DNA2 was a key gene, and high DNA2 expression was found to indicate poor prognosis in 7 of 21 studies (HR > 1, *p* < 0.1). Further, the data from “KM plotter” confirmed that, for thousands of breast cancer patients, high mRNA level of DNA2 was correlated to worse OS, worse RFS, worse DMFS, and worse PPS. All the data strongly suggests that high expression of DNA2 is correlated with worse outcome in breast cancer.

At present, the classic markers of the treatment regimen and prognosis for breast cancer patients are ER and HER2. Breast cancer is commonly separated into Luminal A (LumA), Luminal B (LumB), epidermal growth factor receptor ERBB2/HER2-overexpressing (HER2+), and basal epithelial-like (BL) based on gene expression profiles [5]. LumA and LumB breast cancers are both ER-positive, while LumB breast cancer expresses high proliferation marker Ki67 and low PR, which correlates with a worse prognosis. HER2+ breast cancers overexpress ERBB2/HER2. BL breast cancers lose the expression of ER, PR, or HER2, like triple-negative breast cancers (TNBCs) [22]. We analyzed 2,509 breast cancers in “cBioportal” and 3,063 breast cancer data in “bc-GenExMiner,” respectively, and got the following conclusion: high expression of DNA2 was closely correlated with ER-, PR-, and HER2+, indicating worse prognosis and therapy effect in breast cancer. Further, we validated the above conclusions by immunohistochemistry. The results of the analysis in “bc-GenExMiner” and “cBioportal” showed DNA2 expression in groups with low risk, which are Luminal A (LumA) and Luminal B (LumB) breast cancer, was downregulated. All the results indicated that DNA2 might be a perfect marker of breast cancer prognosis.

We also predicted the potential mechanism of DNA2 action in breast cancer using functional and pathway enrichment analysis. The results showed that high DNA2 expression was related to breast cancer cell cycle, DNA replication, homologous recombination, mismatch repair, nucleotide excision repair, and base excision repair pathways. Also, silencing DNA2 inhibited cancer cell proliferation. These findings are consistent with the previously reported roles of DNA2 in both DNA replication and repair. Collectively, our results indicated that the potential mechanism of DNA2 action in breast cancer might be regulating the cell cycle, DNA replication, homologous recombination, mismatch repair, nucleotide excision repair, and base excision repair pathways.

DNA2 has an important role in protecting the genomic stability. Loss or mutations of DNA2 may inhibit DNA repair pathways in cells. Our results showed that high expression of DNA2 in breast cancer predicted poor prognosis. As mentioned in studies, DNA2 high expression not only occurred in breast and ovarian cancers, but also in liver cancer, testis cancer, thyroid cancer, endometrial cancer, carcinoid cancer, breast cancer, melanoma, and ovarian cancer [12]. DNA2 is important for completing DNA replication and plays a significant role in DNA repair in general. Thus, targeting DNA2 by small molecules is an important strategy to develop treatment modalities. Liu et al. described a chemical inhibitor of DNA2 activities can sensitize cancer cells to chemotherapies [23]. Research also found that chemical inhibition of DNA2 selectively attenuated the growth of various cancer cells [15]. However, the underlying mechanism of DNA2 in breast cancer and subtypes still needs to be defined and be helpful to develop new drugs for cancer therapy.

## Figures and Tables

**Table 1:** Gene sets enriched in the high DNA2 expression phenotype.

Gene set name	NES	NOM *p*-val	FDR *q*-val
KEGG_CELL_CYCLE	2.5039673	0	0
KEGG_OOCYTE_MEIOSIS	2.5004425	0	0
KEGG_PROGESTERONE_MEDIATED_OOCYTE_MATURATION	2.2601042	0	7.58E-04
KEGG_BASAL_TRANSCRIPTION_FACTORS	2.2252226	0	9.09E-04
KEGG_RNA_DEGRADATION	2.1981418	0	9.48E-04
KEGG_SPLICEOSOME	2.1785731	0	9.70E-04
KEGG_DNA_REPLICATION	2.1734233	0	8.31E-04
KEGG_UBIQUITIN_MEDIATED_PROTEOLYSIS	2.0631318	0.001960784	0.00491828
KEGG_HOMOLOGOUS_RECOMBINATION	2.061698	0	0.004467648
KEGG_MISMATCH_REPAIR	2.0155804	0	0.0078261
KEGG_PYRIMIDINE_METABOLISM	1.9722291	0	0.01263749
KEGG_NUCLEOTIDE_EXCISION_REPAIR	1.9698429	0.006237006	0.011753452
KEGG_BASE_EXCISION_REPAIR	1.9483268	0	0.013909859
KEGG_P53_SIGNALING_PATHWAY	1.9202973	0.002020202	0.017711155
KEGG_AMINOACYL_TRNA_BIOSYNTHESIS	1.91081	0.004264392	0.018946456
KEGG_PURINE_METABOLISM	1.9019014	0	0.019136654
KEGG_CYSTEINE_AND_METHIONINE_METABOLISM	1.8780447	0.004	0.022393785
KEGG_LYSINE_DEGRADATION	1.8466676	0.003944773	0.029533371
KEGG_ONE_CARBON_POOL_BY_FOLATE	1.8179266	0.008	0.035526518

NES: normalized enrichment score; NOM: nominal; FDR: false discovery rate. Gene sets with NOM *p* value <0.05 and FDR *q* value <0.05 were regarded as significantly enriched.

## Data Availability

The data used to support the findings of this study have been deposited in “cBioportal” and “bc-GenExMiner.”

## Supplementary Materials

Supplementary Materials Supplementary Table 1 The clinicopathological parameters of cases used in IHC. Supplementary Figure 1. The DNA2 expression exhibited a distinct pattern in breast cancer subtypes. A. The statistic chart showed DNA2 expression among Sorlie's subtypes of breast cancer from the analysis results from bc-GenExMiner. **B.**The statistic chart showed DNA2 expression among breast cancer subtypes from the analysis results from cBioPortal.
