# Supplementary material for: Increased Expression of DNA2 Was Linked to Poor Prognosis in Breast Cancer
Source: Dis Markers. 2021 Jan 26;2021:8860728. doi: 10.1155/2021/8860728 (PMC7857872; doi:10.1155/2021/8860728)
Supplement: Supplementary Materials — Supplementary Table 1 The clinicopathological parameters of cases used in IHC. Supplementary Figure 1. The DNA2 expression exhibited a distinct pattern in breast cancer subtypes. A. The statistic chart showed DNA2 expression among Sorlie's subtypes of breast cancer from the analysis results from bc-GenExMiner. B.The statistic chart showed DNA2 expression among breast cancer subtypes from the analysis results from cBioPortal. [file 8860728.f1.docx]

**Supplemental Information**

**Increased Expression of DNA2 was Linked to Poor Prognosis in Breast cancer**

Yingyan Han^1^, Zeyu Zhang^1^, Zhi Wang^1^, Shujuan Sun^1^*

Supplementary description:

**Supplementary Table 1 The clinicopathological parameters of cases used in IHC.**

**Supplementary Figure 1. The DNA2 expression exhibited a distinct pattern in breast cancer subtypes. A.** The statistic chart showed DNA2 expression among Sorlie’s subtypes of breast cancer from the analysis results from bc-GenExMiner. **B.**The statistic chart showed DNA2 expression among breast cancer subtypes from the analysis results from cBioPortal

**Table S1**

|  |
| --- |
| \| **Clinical characteristics** \| \| \| \| \| --- \| --- \| --- \| --- \| \| **Breast malignant tumor** \| Gender \| Female \| 26 \| \| \| Age \| ≤50 \| 12 \| \| >50 \| 14 \| \| Pathology \| Intraductal carcinoma \| 26 \| \| Histological grade \| I \| 3 \| \| II \| 16 \| \| III \| 4 \| \| ER \| Negative \| 13 \| \| Positive \| 13 \| \| PR \| Negative \| 13 \| \| Positive \| 13 \| \| HER2 \| Negative \| 7 \| \| Positive \| 19 \| \| Lymph node \| Negative \| 13 \| \| Positive \| 13 \| \| **Breast benign tumor** \| Gender \| Female \| 7 \| \| \| Age \| <50 \| 3 \| \| ≥50 \| 7 \| |

**Supplementary Table 1 The clinicopathological parameters of cases used in IHC.**

**Figure S1**


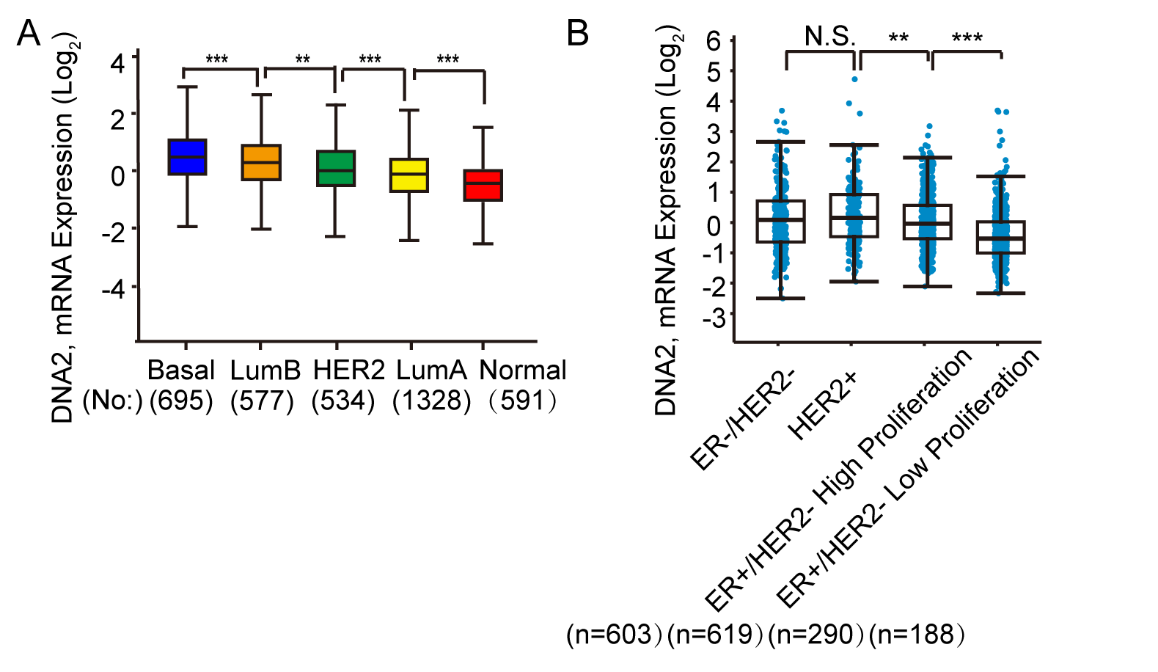


**Supplementary Figure 1. The DNA2 expression exhibited a distinct pattern in breast cancer subtypes. A.** The statistic chart showed DNA2 expression among Sorlie’s subtypes of breast cancer from the analysis results from bc-GenExMiner. **B.**The statistic chart showed DNA2 expression among breast cancer subtypes from the analysis results from cBioPortal.
